# Pea-Derived Protein Supplementation in Sports and Exercise Nutrition: A Narrative Review of Muscle Protein Synthesis and Training Adaptations

**DOI:** 10.3390/nu18213612

**Published:** 2026-10-30

**Authors:** Alice Fognani, Gilda Aiello, Claudia Abate, Wenze Wu, Sara Baldelli, Mauro Lombardo

**Affiliations:** 1 Department of Human Sciences and Quality of Life Promotion, San Raffaele Open University, 00166 Rome, Italy; alice.fognani@uniroma5.it (A.F.);; 2 Inner Mongolia Key Laboratory of Life Health and Bioinformatics, School of Life Science and Technology, Inner Mongolia University of Science and Technology, Baotou 014010, China; 3 College of Life and Health Sciences, Northeastern University, Shenyang 110819, China; 4 IRCCS San Raffaele Roma, Via di Val Cannuta 247, 00166 Rome, Italy

**Keywords:** pea protein, resistance training, sports nutrition, muscle protein synthesis, plant-based protein, strength, body composition

## Abstract

**Background:** Plant-derived proteins are increasingly prominent in sports nutrition, yet pea protein (*Pisum sativum*) is often confounded with multi-ingredient blends or fortified formulations. This narrative review evaluates human and preclinical evidence on pea protein in skeletal muscle anabolism, exercise recovery, and training adaptations. **Methods:** MEDLINE, Scopus, and Web of Science were searched up to September 2026. Thirty-three studies (24 human, 8 animal, 1 in vitro) were stratified by experimental model, intervention directness (isolated pea protein vs. blends/adjuncts), and physiological endpoints. **Results:** Isolated pea protein stimulates postprandial muscle protein synthesis (MPS) under specific conditions, despite lower early peak leucine and essential amino acid concentrations than whey. Longitudinal resistance-training trials report improvements in muscle mass and strength with pea protein; however, available data do not establish an added benefit over training alone or formal equivalence/non-inferiority to whey. Preclinical models suggest potential anti-atrophic bioactivity of specific pea peptides. **Conclusions:** Isolated pea protein supports postprandial MPS and resistance-training adaptations. However, current evidence is insufficient to establish equivalence to dairy proteins. Powered trials with prespecified non-inferiority margins are needed.

## 1. Introduction

In recent years, interest in alternative dietary protein sources has increased substantially within sports and exercise nutrition [1]. Whey protein remains one of the most extensively investigated protein sources in relation to skeletal muscle anabolism because of its high essential amino acid (EAA) and leucine content, favorable postprandial amino acid kinetics, and well-established capacity to stimulate postprandial muscle protein synthesis (MPS) [2,3]. Plant-derived proteins differ considerably in their nutritional characteristics, frequently presenting lower concentrations of one or more EAAs, reduced digestibility, or distinct postprandial amino acid availability [4,5,6]. Pea protein (*Pisum sativum* L.) has emerged as a prominent alternative, yet direct compositional comparisons confirm lower EAA, leucine, and methionine contents relative to dairy proteins [7]. Consequently, evaluating its physiological efficacy requires isolating the intrinsic properties of single-source pea protein from the effects of compensatory strategies, such as increased total protein dosing, multi-source blending, or amino acid fortification [8,9,10,11,12].

The current evidence base investigating pea-derived proteins is characterized by substantial methodological heterogeneity [13]. Although previous systematic and narrative reviews have evaluated plant proteins broadly or addressed pea protein supplementation [13,14,15], existing syntheses present a critical limitation: they aggregate findings across highly disparate intervention models, conflating single-source intact pea protein with multi-ingredient blends, leucine-fortified matrices, hydrolysates, and digestive adjuncts. Crucially, previous reviews have not established whether the observed anabolic responses, recovery kinetics, or long-term training adaptations stem intrinsically from pea protein itself or are driven by confounding variables—such as co-ingested protein sources, elevated total daily protein intakes, or specific processing modifications. Furthermore, existing syntheses frequently equate non-significant trial comparisons with clinical equivalence without evaluating the statistical power of the studies or prespecified equivalence margins.

To address this gap, this narrative review aims to evaluate the evidence concerning pea-derived proteins in relation to muscle protein synthesis, exercise recovery, and resistance-training adaptations by systematically stratifying studies according to experimental model and intervention directness. By distinguishing direct human evidence on isolated pea protein from supportive evidence on multi-source blends and preclinical mechanistic models, this review clarifies which physiological effects are specifically attributable to pea protein and defines the limits of their practical application.

## 2. Materials and Methods

### 2.1. Study Design and Literature Search

This study was designed as a structured narrative review of human, preclinical, and in vitro evidence concerning pea-derived proteins and skeletal muscle metabolism. The review considered studies addressing MPS, postprandial amino acid responses, anabolic signaling, resistance-training adaptations, and selected exercise-recovery outcomes.

Searches of MEDLINE via PubMed, Scopus, and Web of Science were initiated in August 2026 and completed on 30 September 2026. Records published up to 30 September 2026 were eligible for inclusion. Database-specific search syntax was applied as follows:

MEDLINE (via PubMed): (“pea protein”[Title/Abstract] OR “pea-derived protein”[Title/Abstract] OR “pea peptide”[Title/Abstract] OR “Pisum sativum”[Title/Abstract]) AND (“resistance training”[Title/Abstract] OR “muscle protein synthesis”[Title/Abstract] OR “muscle hypertrophy”[Title/Abstract] OR “exercise recovery”[Title/Abstract] OR “muscle damage”[Title/Abstract] OR “strength”[Title/Abstract] OR “physical performance”[Title/Abstract] OR “muscle atrophy”[Title/Abstract] OR “amino acid availability”[Title/Abstract] OR “mTORC1”[Title/Abstract]).

SCOPUS: TITLE-ABS-KEY((“pea protein” OR “pea-derived protein” OR “pea peptide” OR “Pisum sativum”) AND (“resistance training” OR “muscle protein synthesis” OR “muscle hypertrophy” OR “exercise recovery” OR “muscle damage” OR “strength” OR “physical performance” OR “muscle atrophy” OR “amino acid availability” OR “mTORC1”)).

Web of Science (Core Collection): TS = ((“pea protein” OR “pea-derived protein” OR “pea peptide” OR “Pisum sativum”) AND (“resistance training” OR “muscle protein synthesis” OR “muscle hypertrophy” OR “exercise recovery” OR “muscle damage” OR “strength” OR “physical performance” OR “muscle atrophy” OR “amino acid availability” OR “mTORC1”)).

Eligibility was restricted to English-language peer-reviewed articles, with one exception retained during revision: a Persian-language report with an extended English summary relevant to exercise recovery. Grey literature was not searched.

### 2.2. Eligibility Criteria

Eligible studies included those evaluating isolated pea protein, pea-protein concentrates, pea-containing blends, pea-derived peptides or hydrolysates, or formulations in which pea protein represented a clearly defined component. Eligible outcomes encompassed acute muscle protein synthesis (MPS), postprandial amino acid kinetics, anabolic signaling, exercise recovery markers, and longitudinal resistance-training adaptations (e.g., muscle mass, thickness, cross-sectional area, strength, and physical performance). Studies focusing exclusively on technological, sensory, agricultural, or food-processing characteristics without muscle-related physiological outcomes were excluded.

To structure the narrative synthesis, evidence was stratified across three distinct streams based on experimental model and intervention directness: (1) direct human evidence, comprising human trials evaluating intact, single-source pea protein isolates or concentrates as the sole intervention protein source; (2) supportive human evidence, encompassing human trials examining multi-source plant blends, amino acid-fortified formulations, digestive adjuncts, or pea-derived peptides and hydrolysates where the independent contribution of pea protein could not be isolated; and (3) preclinical and in vitro mechanistic evidence, including animal models and cell culture experiments, regardless of formulation, used strictly to contextualize potential biological mechanisms rather than infer clinical efficacy in humans.

Human trials were further classified by study design into longitudinal training interventions (combining protein supplementation with structured exercise) and acute or short-term physiological studies (assessing postprandial aminoacidemia, MPS, or recovery kinetics). For studies reporting mixed endpoints, each specific outcome was evaluated separately within its corresponding physiological category.

### 2.3. Study Selection and Data Extraction

Titles, abstracts, and full-text articles were screened for eligibility as summarized in Supplementary Figure S1. Extracted data parameters included study design, experimental model, participant characteristics, sample size, training status, protein source and formulation, protein dose, comparator, protocol duration, exercise conditions, background dietary protein and essential amino acid intake, outcome measures, and principal findings. Numerical results are presented using summary statistics (mean ± SD) and *p* values as reported in original publications; no quantitative meta-analysis or statistical reanalysis was performed due to substantial clinical and methodological heterogeneity.

No formal quantitative risk-of-bias tool (e.g., RoB 2 or ROBINS-I) or formal quality scoring matrix was applied, which represents an inherent limitation of this narrative review. Instead, a qualitative critical appraisal approach was integrated directly into the narrative synthesis and discussion. Seven core methodological domains—specifically (1) statistical power and sample size, (2) protocol duration, (3) participant training status, (4) background dietary protein intake, (5) comparator selection, (6) formulation directness, and (7) outcome directness—were applied as narrative critical appraisal criteria to contextualize the strength of evidence and methodological limitations of each included study within their respective sections.

### 2.4. Narrative Synthesis

Given the substantial clinical, methodological, and experimental heterogeneity across studies, the evidence was synthesized narratively. Longitudinal training studies, acute and short-term mechanistic studies, exercise-recovery studies, and preclinical or in vitro investigations were considered separately.

**Figure 1:**
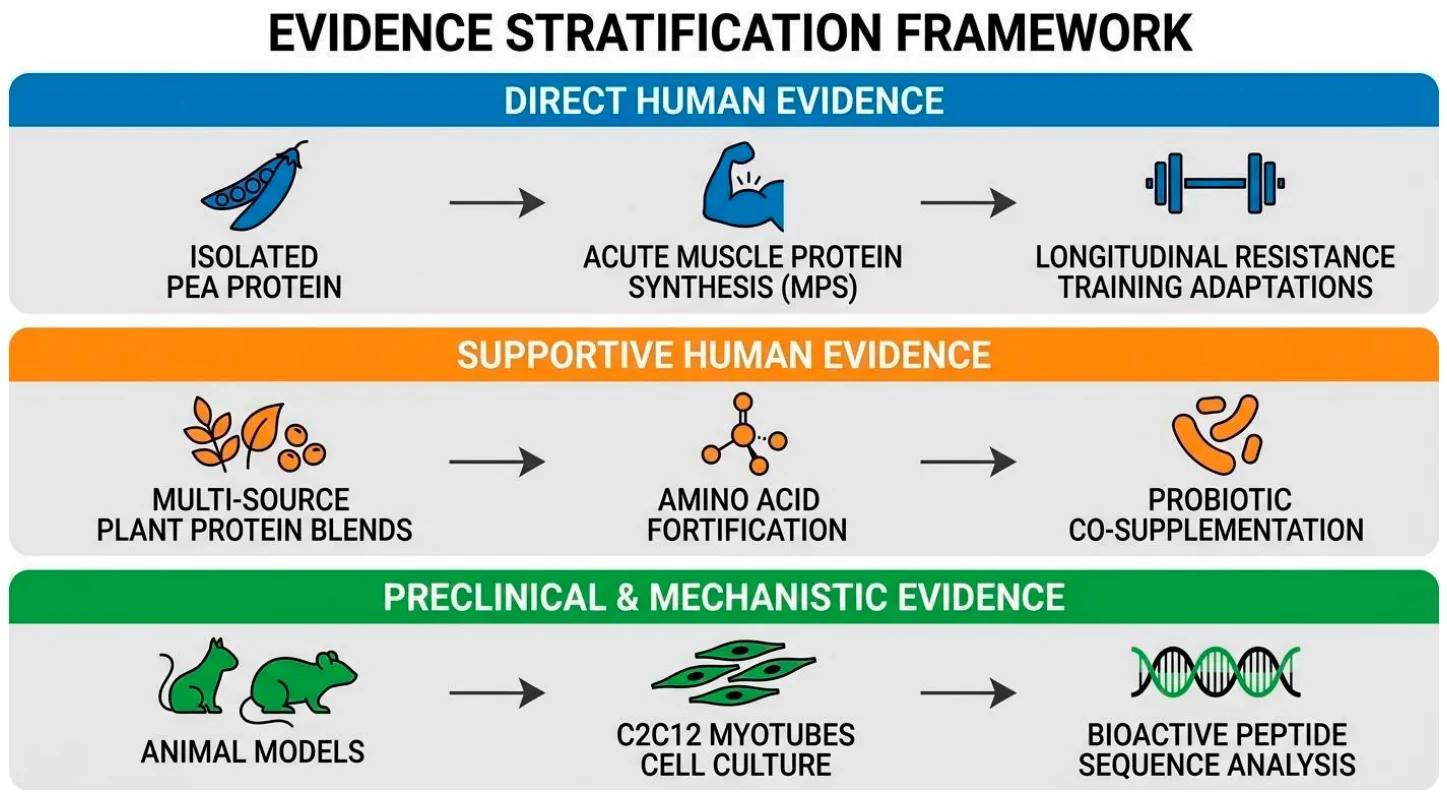
Methodological framework for the stratification of evidence on pea-derived protein in sports and exercise nutrition. Studies are classified across three distinct tiers based on experimental directness: (1) Direct Human Evidence (isolated single-source pea protein evaluating acute muscle protein synthesis, postprandial kinetics, and resistance-training adaptations); (2) Supportive Human Evidence (multi-source plant blends, amino acid fortification, and adjunctive ingredients); and (3) Preclinical and Mechanistic Evidence (animal models, cell cultures, and bioactive peptides). These categories describe evidence sources and do not imply a causal sequence from acute MPS to training adaptations.

The synthesis emphasized the distinction between single-source pea-derived proteins and pea-containing multi-source formulations, direct and supportive human evidence, human and preclinical evidence, acute physiological responses and longer-term training adaptations, and direct MPS measurements and indirect outcomes such as amino acid concentrations or anabolic signaling. The methodological stratification framework classifying evidence by experimental directness and physiological endpoints is illustrated in Figure 1.

## 3. Results

A total of 133 articles were assessed in full text, of which 33 publications were ultimately included in the narrative synthesis, comprising 24 human reports, 8 animal studies, and 1 in vitro study [7,8,9,10,11,12,16,17,18,19,20,21,22,23,24,25,26,27,28,29,30,31,32,33,34,35,36,37,38,39,40,41,42]. The two Korzepa reports [18,23] share trial registration NCT05574205 and report different outcomes from the same trial. They are counted as two publications rather than two independent cohorts; sample sizes vary by outcome.

### 3.1. Longitudinal Resistance-Training Studies

Human longitudinal evidence specifically investigating pea protein supplementation during resistance or high-intensity functional training remains relatively limited. Available studies were conducted in populations differing in training status and used heterogeneous supplementation protocols. Direct comparisons primarily involved single-source pea-derived and whey proteins, whereas other investigations assessed pea-containing protein blends or pea protein combined with adjunctive ingredients intended to modify protein utilization.

Babault et al. [8] randomized 161 untrained men to pea protein, whey protein, or placebo during a 12-week resistance-training program; 137 participants were included in the final analysis. Participants assigned to the protein groups consumed 25 g twice daily, corresponding to 50 g/day. Biceps brachii muscle thickness increased over time in all groups, but neither a significant overall group effect nor a group-by-time interaction was detected. Changes from baseline to week 12 were +13.4 ± 10.8% with pea protein, +15.3 ± 12.7% with whey protein, and +10.7 ± 8.6% with placebo, with the overall between-group comparison not reaching statistical significance (*p* = 0.09). Thus, an independent supplementary benefit of pea protein over placebo was not established in the primary analysis. In a sensitivity analysis restricted to participants with a baseline 1-RM < 25 kg, increases were +20.2 ± 12.3% with pea protein, +15.6 ± 13.5% with whey protein, and +8.6 ± 7.3% with placebo. The pea–placebo comparison was significant, whereas neither the pea–whey nor the whey–placebo comparison was significant. This subgroup result should be interpreted cautiously alongside the non-significant overall group-by-time interaction.

Banaszek et al. [9] compared pea and whey protein supplementation during 8 weeks of high-intensity functional training in 15 resistance-trained men and women. Participants consumed approximately 49 g/day of either pea or whey protein. Reported total protein intake was 1.7 ± 0.4 g/kg/day in the pea group and 1.8 ± 0.3 g/kg/day in the whey group. Both groups improved 1-RM squat and deadlift performance, with no significant between-group differences. No significant changes or between-group differences were observed for body composition, muscle thickness, isometric mid-thigh pull performance, or workout performance.

Singh et al. [10] evaluated pea vs. whey protein supplementation during an 84-day resistance-training program in sedentary adults. Depending on flavor, the pea supplement provided approximately 20.0–22.5 g protein/day, whereas the whey comparator provided 20.0–21.1 g/day. Of the 50 participants randomized to the pea and whey groups, 35 were included in the per-protocol analysis (18 pea; 17 whey). Composite whole-body muscle strength increased by 16.1% from baseline in the pea group (*p* = 0.01) and by 11.1% in the whey group (*p* = 0.06). However, no significant between-group differences were detected for composite whole-body muscle strength or individual strength outcomes. The different within-group *p* values do not establish a greater effect of pea protein than whey protein. Body-composition outcomes likewise showed no significant differential effect of protein source.

Lee et al. [16] investigated whether probiotic co-supplementation could modify the physiological response to pea protein during resistance training. Thirty-two adults (16 men and 16 women) were randomized to receive 20 g/day of pea protein either alone or combined with 1 × 10^10^ colony-forming units of *Lactiplantibacillus plantarum* TWK10 during 28 days of resistance exercise training performed three times per week. Compared with pea protein alone, the TWK10-supplemented group showed greater increases in muscle thickness and improvements in 1-RM bench-press performance, explosive power, and anaerobic performance. The probiotic-supplemented condition also produced greater postprandial exposure and peak concentrations of BCAAs, EAAs, and total amino acids. Because both groups received the same dose of pea protein, however, this study primarily informs the potential effect of probiotic co-supplementation on pea-protein utilization rather than the independent efficacy of pea protein itself.

More recently, Santini et al. [17] investigated a pea-containing plant-protein formulation during a 12-week supervised resistance-training program. Of 52 randomized participants, 44 completed the intervention (22 per group). The protocol specified a nominal protein dose of 45 g/day, divided into three daily servings, provided as either a soy–pea protein blend or whey protein. Mean total protein intake during supplementation was approximately 1.7–1.8 g/kg/day in the plant-protein group and 1.8 g/kg/day in the whey group. Whole-body lean mass increased from 50.21 ± 4.78 to 52.62 ± 4.37 kg in the plant-protein group, corresponding to an increase of approximately 2.41 kg, and from 49.08 ± 4.97 to 52.31 ± 5.19 kg in the whey group, corresponding to approximately 3.23 kg. Significant effects of time were observed for whole-body, appendicular, and leg lean mass, without significant group-by-time interactions. A similar pattern was observed for muscle cross-sectional area and strength-related outcomes. Because the plant-based supplement contained both soy and pea proteins, these findings provide supportive evidence for pea-containing protein blends rather than direct evidence regarding isolated pea protein.

Overall, longitudinal studies indicate that single-source pea-derived protein and pea-containing formulations can be incorporated into nutritional strategies used alongside resistance training and can accompany improvements in muscle mass, muscle thickness, strength, and performance-related outcomes. However, the available evidence remains insufficient to establish superiority or formal equivalence relative to whey protein. Direct comparative trials are few, sample sizes are generally modest, intervention durations are relatively short, and studies differ substantially in training status, protein dose, formulation, and accompanying dietary conditions. Furthermore, findings obtained with multi-source blends or adjunctive probiotic formulations should not be attributed specifically to pea protein when its independent contribution cannot be isolated. Accordingly, the absence of statistically significant between-group differences in several trials should not be interpreted as evidence of formal equivalence. Table 1 shows the detailed characteristics of the studies included in this section.

### 3.2. Acute and Integrated Muscle Protein Synthesis

Human mechanistic studies provide complementary evidence regarding the capacity of pea-derived proteins to stimulate MPS. These investigations differ from longitudinal training trials because they primarily assess acute postprandial or post-exercise responses or integrated MPS over relatively short supplementation periods. Accordingly, their findings inform acute or short-term physiological responses rather than longer-term hypertrophy or training adaptation.

McKendry et al. [12] examined integrated myofibrillar protein synthesis during sequential 7-day dietary phases in older men. Forty-five participants were randomized, and 31 completed the study (11 pea; 10 whey; 10 collagen). Participants initially consumed protein at approximately the recommended dietary allowance and subsequently received an additional 25 g of pea, whey, or collagen protein twice daily. Each 25-g protein serving supplied 9.78 g of EAAs and 2.06 g of leucine in the pea condition, compared with 12.50 g of EAAs and 2.70 g of leucine in the whey condition. Increasing protein intake to approximately 1.3 g/kg/day increased integrated MPS in the pea- and whey-supplemented groups, with the same observed mean supplementation-phase rates of approximately 1.59%/day in both groups, whereas collagen did not produce the same increase. Importantly, this study did not include a resistance-training intervention and therefore informs the capacity of pea protein to support integrated protein synthesis rather than training-induced hypertrophy.

Korzepa et al. [18] extended this evidence by investigating daily integrated myofibrillar protein synthesis during a 10-day controlled dietary intervention in 27 middle-to-older-aged adults. This report and the report on postprandial aminoacidemia [23] derive from the same registered trial, NCT05574205, with sample sizes differing by outcome. Participants consumed approximately 1.0 g protein/kg body mass/day from either an animal-focused diet supplemented with whey protein (*n* = 14) or a plant-focused diet supplemented with pea protein (*n* = 13), while performing supervised unilateral knee-extension resistance training every other day. Daily integrated myofibrillar protein synthesis was significantly greater in the trained than in the untrained leg in both dietary conditions, reaching 1.44 ± 0.26 vs. 1.29 ± 0.27%·day^−1^ in the whey-supplemented diet and 1.50 ± 0.17 vs. 1.34 ± 0.21%·day^−1^ in the pea-supplemented diet (*p* < 0.001). No significant group-by-training interaction (*p* = 0.956) or main effect of dietary group (*p* = 0.52) was detected. Because the intervention involved broader differences in dietary protein-source composition in addition to the supplemental protein, these findings provide supportive evidence for a pea-protein-supplemented, plant-focused dietary pattern rather than a direct comparison between isolated pea and whey proteins.

In resistance-trained individuals, West et al. [11] examined the acute post-exercise MPS response to 25 g of pea protein, mycoprotein, or a blend of the two. Thirty-three participants completed a whole-body resistance-exercise bout before protein ingestion. Myofibrillar fractional synthetic rates increased from postabsorptive values in all conditions and reached 0.087 ± 0.010%·h^−1^ following pea protein, 0.076 ± 0.004%·h^−1^ following mycoprotein, and 0.085 ± 0.010%·h^−1^ following the blend, with no significant differences among conditions.

Pinckaers et al. [7] directly compared 30 g of pea-derived protein with an equivalent amount of milk-derived protein in 24 healthy young men using a randomized, double-blind, parallel-group design. The pea-protein dose provided 7.7 g of EAAs and 1.8 g of leucine, compared with 9.8 g of EAAs and 2.4 g of leucine in the milk-protein condition. Postprandial myofibrillar protein synthesis increased following protein ingestion, and no significant difference in the MPS response was detected between the pea- and milk-protein groups. Because the study assessed a specific acute response in healthy young men, its findings should not be extrapolated directly to other populations or to long-term training adaptations.

Evidence from protein blends provides additional information regarding the influence of amino acid composition. Lim et al. [19] used a randomized crossover design in young men and women to compare 20 g of protein provided as whey, a plant-protein blend containing 88% pea and 12% canola protein, or the same plant-protein blend fortified with leucine to match the leucine content of whey. The unfortified plant-protein blend elicited a lower MPS response than whey, whereas leucine fortification increased the response such that it no longer differed significantly from whey. These findings suggest that leucine availability may influence the acute anabolic response to a predominantly pea-based formulation, but they do not establish that all pea-protein products require leucine fortification or that the fortified formulation is equivalent to whey.

Similarly, van der Heijden et al. [20] compared 32 g of whey protein with an isonitrogenous plant-protein blend containing 39.5% pea, 39.5% brown rice, and 21% canola protein in resistance-trained adults following resistance exercise. Postprandial EAA availability was substantially greater following whey ingestion; nevertheless, post-exercise myofibrillar protein synthesis increased following both protein conditions, without significant between-condition differences during the assessed postprandial periods. Because the intervention was a multi-source plant-protein blend, the result provides evidence regarding the blend as a whole rather than isolated pea protein.

Pinckaers et al. [21] compared 30 g of milk protein with 30 g of an exclusively plant-derived blend containing 15 g wheat, 7.5 g corn, and 7.5 g pea protein in healthy young men. Postprandial MPS did not differ significantly between conditions. Because pea protein accounted for only one-quarter of the plant-protein blend, this study provides supportive evidence for protein-complementation strategies rather than direct evidence regarding isolated pea protein.

Taken together, direct acute human studies indicate that single-source pea-derived protein can stimulate MPS under selected resting and post-exercise conditions. In the 10-day dietary study, MPS was higher in the trained than in the untrained leg under both dietary conditions, with no significant dietary-group effect or group-by-training interaction; this result does not isolate a pea-specific effect. Findings from multi-source diets and protein blends should remain distinct from direct evidence obtained with single-source pea-derived protein (Table 2).

### 3.3. Amino Acid Availability and Anabolic Signaling

Studies examining postprandial circulating amino acid responses provide additional context for the MPS findings. Differences between pea- and animal-derived proteins have been observed even in experimental settings in which clear differences in MPS were not detected. These outcomes should be interpreted as complementary physiological measures and not as direct substitutes for MPS assessment.

Lanng et al. [22] investigated acute amino acid and intracellular signaling responses to pea, whey, or cricket protein in a randomized, single-blind, parallel-group trial involving healthy young men. Following one-legged resistance exercise, participants ingested 0.25 g protein/kg fat-free mass of one of the three protein sources. Whey protein elicited greater early postprandial increases in circulating EAAs, branched-chain amino acids (BCAAs), and leucine than pea protein, with significantly higher concentrations at 20, 40, and 60 min. Incremental area under the curve (iAUC) for EAAs, BCAAs, and leucine was also greater following whey ingestion, whereas total amino acid and EAA AUC did not differ significantly between protein sources. Despite these differences in circulating amino acid responses, no significant effect of protein source was detected for phosphorylation of the assessed mTORC1-related signaling proteins in either the exercised or non-exercised leg 3 h after protein ingestion. Importantly, MPS was not measured directly; therefore, these findings should be interpreted as evidence regarding acute intracellular signaling rather than as evidence of equivalent MPS responses.

Korzepa et al. [23] compared pea protein isolate with whey protein added to a low-protein breakfast in middle-to-older-aged adults. Twenty-seven participants were recruited, and complete plasma amino acid data were available for 22 participants (13 whey; 9 pea). Plasma leucine concentrations were significantly greater following whey at 40 min postprandially, and leucine iAUC was greater with whey (*p* = 0.032). However, peak leucine concentrations did not differ significantly between conditions (*p* = 0.081), nor did peak EAA concentrations or EAA iAUC. These findings therefore indicate differences in leucine kinetics without demonstrating uniformly greater overall postprandial EAA exposure following whey.

Rogers et al. [24] further compared postprandial aminoacidemia following ingestion of pea protein isolate, a pea–rice protein blend, and whey protein concentrate in 10 healthy young adults (four men and six women) using a randomized crossover design. Participants ingested 0.3 g protein/kg body mass in each condition. Plasma EAA iAUC over 4 h was significantly greater following whey than following pea protein (*p* = 0.011) or the pea–rice blend (*p* = 0.014), whereas no significant difference was observed between pea protein and the blend (*p* = 0.318). Peak plasma leucine concentrations were likewise greater after whey than after pea protein or the pea–rice blend, with no significant difference between the two plant-protein conditions. Thus, under the conditions tested, combining pea and rice protein did not normalize the postprandial EAA or leucine response to that observed with whey.

In healthy older adults, de Marco Castro et al. [25] compared whey protein with three plant-protein–fiber formulations containing pea protein in different proportions. The plant formulations were designed to provide an optimized amino acid profile and were matched for approximately 2.8 g of leucine. Despite this matching, peripheral postprandial amino acid appearance was lower following the plant-protein products than following whey. Because pea protein constituted only one component of these formulations, the findings should be interpreted as supportive evidence regarding pea-containing protein matrices rather than as a direct comparison between isolated pea and whey protein.

Salles et al. [26] investigated postprandial amino acid responses following pea or whey protein ingestion in 30 healthy men, comprising 15 younger adults aged 20–28 years and 15 older adults aged 65–81 years. Participants received pea or whey protein either alone or following a standardized meal in a randomized crossover design. The protein solution provided 0.41 g protein/kg body weight, while the complete meal condition provided a total of 0.54 g/kg. Postprandial amino acid profiles differed according to protein source, age, and feeding condition. Whey protein produced a greater leucine response, whereas pea protein produced greater circulating arginine concentrations. Plasma collected at each participant’s peak leucine concentration was subsequently used to condition C2C12 myotubes. Despite differences in circulating amino acid profiles, the resulting effects on myotube protein synthesis were age-dependent but not significantly dependent on protein source. Because skeletal-muscle MPS was not measured directly in the participants, this hybrid human–in vitro design provides mechanistic evidence rather than direct evidence of comparative human MPS.

Paulussen et al. [27] investigated whether microbial protease co-ingestion could modify postprandial amino acid availability following pea-protein ingestion in 24 healthy adults using a randomized, double-blind crossover design. Participants consumed 25 g of pea protein powder (providing 20 g of intact protein) with either a mixture of three microbial proteases or placebo. Protease co-ingestion significantly increased early (0–2 h) net plasma exposure to leucine, BCAAs, EAAs, and total amino acids. In contrast, leucine, BCAA, and EAA exposure did not differ significantly between conditions across the complete 5-h postprandial period. These findings indicate that protease supplementation can modify the early postprandial amino acid response to pea protein but do not establish an enhanced anabolic response because skeletal-muscle MPS was not measured.

Rathi et al. [28] similarly investigated whether an enzyme–probiotic formulation could modify amino acid responses to pea protein in 15 healthy adults aged 18–35 years using a randomized, double-blind, placebo-controlled crossover design. Participants consumed 30 g/day of pea protein with either an enzyme–probiotic blend or maltodextrin placebo during two 15-day supplementation periods separated by a washout period. Postprandial amino acid responses were similar between conditions on day 1. By day 15, the enzyme–probiotic condition showed numerically greater concentrations of several amino acids, including leucine, isoleucine, lysine, methionine, threonine, and tryptophan; however, the increases in overall EAA and BCAA responses were not statistically significant. These findings should therefore be considered exploratory evidence regarding potential modification of pea-protein digestion and amino acid appearance rather than evidence of increased muscle anabolism.

Overall, studies of postprandial amino acid responses indicate that pea-derived proteins can produce substantial increases in circulating amino acids, although whey protein frequently elicits a greater early leucine and EAA response. Formulation effects were not consistent across studies. The pea–rice blend did not improve EAA iAUC or peak leucine concentrations relative to pea protein alone, whereas enzyme or probiotic co-supplementation modified selected amino acid responses in some studies. These interventions did not establish an increase in MPS. However, greater circulating amino acid concentrations do not necessarily translate proportionally into greater MPS, as illustrated by studies in which differences in aminoacidemia were observed without corresponding differences in MPS or anabolic signaling. Accordingly, circulating amino acid responses, intracellular signaling, and directly measured skeletal-muscle protein synthesis should remain conceptually distinct outcomes when interpreting the anabolic potential of pea-derived proteins (Table 2).

### 3.4. Exercise Recovery Outcomes

Evidence regarding exercise recovery is more limited and does not necessarily parallel findings for MPS or longer-term training adaptation.

Coutinho et al. [29] evaluated acute recovery following a strenuous military exercise protocol in 24 Brazilian Army soldiers allocated to carbohydrate alone, a higher-carbohydrate condition, or carbohydrate combined with pea protein concentrate. The carbohydrate-plus-protein group received 0.8 g carbohydrate/kg body mass/h together with 0.2 g pea protein/kg body mass/h immediately and at 60 and 120 min after exercise. Creatine kinase concentrations decreased over the subsequent 24 h in all groups, whereas maximal isometric strength and lactate dehydrogenase did not differ significantly across assessment times. Importantly, adding pea protein to carbohydrate did not improve muscle-function recovery or attenuate biochemical markers of muscle damage compared with carbohydrate alone. Because pea protein was co-ingested with carbohydrate and was not tested as an isolated intervention, this study provides supportive rather than direct evidence.

Nieman et al. [30] randomized 92 non-athletic, non-obese men to pea protein, whey protein, or a water-only control during a 5-day recovery period following a 90-min whole-body eccentric exercise bout. Pea and whey protein were provided in three daily doses of 0.3 g/kg (0.9 g/kg/day), with a single 0.3 g/kg dose on the final morning. Whey protein significantly attenuated post-exercise increases in serum creatine kinase and myoglobin compared with water. Pea protein produced intermediate but non-significant effects, and no significant differences were reported between pea and whey protein. Neither protein supplement meaningfully altered delayed-onset muscle soreness or exercise-performance outcomes.

Hosseinzade et al. [31] compared pea protein isolate, whey protein isolate, and placebo in 30 healthy young men following a 60-min high-intensity functional training session. Participants received 0.3 g protein/kg body mass before and again after the exercise bout, and an additional serving was provided for consumption before bedtime. Significant time, group, and time-by-group effects were reported for delayed-onset muscle soreness (DOMS), creatine kinase, and lactate dehydrogenase. Both protein-supplemented groups showed more favorable responses than placebo for several muscle-damage outcomes, while whey protein produced a greater attenuation of DOMS than pea protein. No clear corresponding advantage was observed for the physical-fitness outcomes assessed. These findings suggest that pea protein may attenuate selected indices of exercise-induced muscle damage relative to placebo, although the response was not uniformly comparable with whey.

Spoelder et al. [32] examined 13 days of supplementation with 25 g/day pea protein, whey protein, or placebo in 47 physically active older adults undergoing prolonged walking exercise. Whey protein attenuated the exercise-induced increase in creatine kinase at 24 h compared with both placebo and pea protein. In contrast, no significant between-group differences were observed for lactate dehydrogenase, muscle soreness, muscle strength, or skeletal muscle mass. These findings suggest that the response of biochemical markers of muscle damage may differ according to protein source, while functional recovery outcomes may remain unaffected.

Loureiro et al. [33] compared pea protein concentrate with whey protein during recovery from a simulated soccer match in 12 male U-20 soccer players using a randomized, double-blind crossover design. Participants consumed 0.5 g protein/kg body mass from pea or whey protein during 10-day supplementation periods. Countermovement-jump performance and perceived muscle pain did not differ significantly between protein conditions during the 72-h recovery period. However, changes in creatine kinase relative to baseline were lower following pea protein than whey protein immediately after the match and at 24 and 48 h, while lactate responses were also lower immediately post-match in the pea-protein condition. Despite these biochemical differences, multivariate metabolomic analyses showed limited overall separation between the protein conditions. Accordingly, this study suggests that pea and whey protein may differentially influence selected biochemical responses to soccer exercise, but it does not demonstrate superior functional recovery with either protein source.

Taken together, the available recovery studies show heterogeneous and outcome-specific responses. Pea protein has produced favorable changes in selected biochemical markers relative to placebo or whey under some experimental conditions, whereas other studies have found no advantage over carbohydrate or placebo, and whey has attenuated selected markers of muscle damage more effectively in other settings. Functional outcomes, including strength, countermovement-jump performance, physical-fitness measures, and perceived muscle soreness, have generally shown fewer or less consistent differences between protein sources. Therefore, changes in creatine kinase, myoglobin, lactate dehydrogenase, or other circulating biomarkers should not be interpreted as direct evidence of improved functional recovery, hypertrophy, or longer-term training adaptation. Differences in age, training status, exercise model, protein dose, and supplementation timing further limit direct comparison among studies (Table 2).

### 3.5. Preclinical and In Vitro Evidence

Preclinical studies provide mechanistic information on pea-derived proteins but vary substantially in experimental model and formulation. The evidence includes one direct long-term comparison of pea protein with dairy-derived proteins, as well as studies of pea protein combined with inulin, complementary plant-protein blends, pea-derived peptides, leucine-fortified formulations, and enzyme-modified or fermented pea protein. Findings from these models were interpreted as mechanistic or supportive evidence and were not used to infer efficacy or equivalence in humans. Table 3 summarizes the characteristics of the studies included in this section.

Salles et al. [34] directly compared pea protein with dairy-derived proteins in 30 20-month-old male Wistar rats fed isocaloric diets containing 14% pea protein, casein, or whey protein for 16 weeks. Body weight, fat mass, and lean mass did not differ significantly among protein groups at the end of the intervention. Similarly, plantaris muscle fractional protein-synthesis rates and p70S6K phosphorylation were not significantly affected by protein source. The study found no statistically significant differences among the protein groups in body composition, plantaris muscle protein-synthesis rates, or p70S6K phosphorylation. These findings do not establish equivalence with casein or whey, and they cannot be extrapolated directly to human skeletal-muscle responses.

Salles et al. [35] investigated old rats fed a diet containing pea protein, either alone or in combination with inulin, for 16 weeks. Compared with pea protein alone, the addition of inulin was associated with improvements in fast-twitch skeletal muscle-related outcomes and indices of mitochondrial activity. These findings support a potential interaction between pea protein and inulin but do not demonstrate an intrinsic advantage of isolated pea protein relative to animal-derived proteins.

Lépine et al. [36] compared a wheat–pea protein blend with milk protein in a rat model and reported that the plant-protein blend supported tissue protein anabolism while producing differences in metabolic regulation. This study provides evidence regarding a complementary plant-protein formulation but does not isolate the specific contribution of pea protein.

Berrazaga et al. [37] investigated mixed wheat–legume pasta products in old rats, including a formulation containing 79% split-pea flour and 21% wheat semolina. MPS in animals receiving mixed wheat–legume products was not significantly different from that observed with casein in several comparisons, although soluble milk proteins produced a stronger anabolic response for some outcomes. Because the intervention consisted of whole-food matrices rather than isolated pea protein, these findings constitute indirect evidence.

Jia et al. [38] examined pea-derived peptides in conjunction with resistance exercise in rats. Daily administration of 0.4 g/kg pea peptides during 8 weeks of resistance exercise increased muscle mass and strength and was accompanied by changes in hypertrophy-related signaling pathways compared with control conditions.

Özdemir et al. [39] examined an acute pea–rice protein formulation in 24 exercised rats. Animals received exercise alone, a 1:1 pea–rice protein blend (0.465 g/kg body weight), or the pea–rice blend combined with an amylopectin/chromium complex after 2 h of exercise. Compared with exercise alone, the pea–rice blend increased MPS by approximately 30.2%, whereas the formulation containing amylopectin/chromium increased MPS by approximately 42.6% (*p* < 0.0001). The fortified formulation also produced greater phosphorylation of mTOR and S6K1 than the pea–rice protein blend alone. Because pea protein was administered together with rice protein and, in one condition, additional amylopectin/chromium, the findings provide mechanistic evidence regarding multi-component formulations rather than the independent anabolic effect of pea protein.

In aged mice, Dijk et al. [40] examined the effect of leucine fortification of a pea–soy protein formulation. The unfortified pea–soy formulation did not significantly increase MPS compared with the fasted condition, whereas the leucine-fortified formulation significantly stimulated MPS and produced a response that did not differ significantly from whey. This murine study supports the potential importance of formulation and leucine content in the experimental model, but the absence of a significant difference from whey does not establish formal equivalence even within the murine model and cannot be extrapolated to humans. These findings further support the potential importance of amino acid composition, particularly leucine content, in determining the anabolic response to plant-protein formulations.

Finally, Kim et al. [41] investigated enzyme-modified and fermented pea protein in dexamethasone-treated C2C12 myotubes. The intervention attenuated molecular markers associated with dexamethasone-induced muscle atrophy and modulated FoxO3-related signaling. This study therefore provides in vitro mechanistic evidence and should be interpreted separately from animal and human studies. Because the model involved dexamethasone-induced atrophy in cultured myotubes, the findings are hypothesis-generating and do not establish an anti-atrophic effect in humans.

#### Pea-Derived Bioactive Peptides: Sequence-Level Evidence and Implications for Skeletal Muscle

Pea-derived peptides provide a mechanistic perspective that complements the predominantly amino acid-centered interpretation of pea protein supplementation. However, evidence obtained with protein hydrolysates must be distinguished from experiments involving chemically defined peptides. Hydrolysates supply heterogeneous mixtures of peptides and amino acids, whereas testing synthetic sequences enables more specific evaluation of biological activity. Current studies support the investigation of pea-derived peptides in muscle cell growth, atrophy-related signaling, and exercise tolerance, although these endpoints differ in their relevance to human muscle adaptation.

Jia et al. [38] combined animal experiments with bioactivity-guided fractionation. Rats receiving pea peptides at 0.4 g/kg/day during eight weeks of resistance training showed improvements in muscle morphology and grip strength. Subsequent analysis of an active chromatographic fraction identified five sequences, EGSLLLPH, LDLPVL, LLYVIR, TNYEEIEKVLL, and NTNYEEIEKVL, which were synthesized and tested in C2C12 cells. All increased the MTT-based proliferation readout after 48 h, with LDLPVL producing the most pronounced response and an effect detectable at 24 h. LDLPVL also increased IGF-1R, cyclin D1, CDK6, and AMPK phosphorylation.

Complementary evidence concerns the regulation of atrophy-associated responses. Kim et al. [41] investigated enzyme-modified and fermented pea protein in dexamethasone-treated C2C12 cells. Following UHPLC–MS/MS profiling, synthetic counterparts of five peptides identified in the pea-derived preparation—ALEPDHRVE, IENPVKEL, IQRPVKEL, IQGITKPAI, and VERPVKEL—were screened. IENPVKEL and IQRPVKEL reduced FoxO3a transcript levels at 10 µg/mL; MuRF-1 expression decreased with IENPVKEL at 5–10 µg/mL, IQRPVKEL at 10 µg/mL, and IQGITKPAI at 5 µg/mL. Further experiments showed increased Sirt1 and AMPK gene expression with IENPVKEL at 5 µg/mL and IQRPVKEL at 2–5 µg/mL.

At the hydrolysate level, Feng et al. [42] reported longer weight-loaded swimming times in mice following 30 days of pea peptide supplementation, accompanied by changes in blood lactate, blood urea nitrogen, glycogen stores, and antioxidant and immune parameters.

## 4. Discussion

The evidence reviewed supports the capacity of intact pea protein to stimulate human MPS under specific experimental conditions [7,11,12]. Its independent contribution to longer-term training adaptations is less clearly established [8,9,10]. These questions require different types of evidence: tracer studies directly assess protein synthesis, whereas longitudinal trials determine whether supplementation affects muscle mass, strength, or performance. Intervention directness and methodological quality must also be considered separately. A study of isolated pea protein permits more specific attribution than a study of a blend, but its conclusions still depend on the comparator, sample, analysis, and outcome.

For training adaptations, the distinction between improvement during supplementation and improvement attributable to supplementation is central. Trials comparing pea with whey without a non-protein control cannot determine how much of the observed improvement reflects training alone [9,10]. Babault et al. [8] included a placebo comparison, but the favorable pea–placebo finding arose from a subgroup analysis alongside a non-significant overall comparison. This provides a narrower basis for inference than a positive primary analysis. The pilot sample in Banaszek et al. [9] and the exclusions from the per-protocol analysis in Singh et al. [10] further limit precision and, in the latter case, raise concerns about the consequences of analyzing a selected subset of randomized participants.

The interpretation of non-significant comparisons also depends on the hypothesis being tested. Failure to detect a difference in a superiority trial does not demonstrate equivalence or non-inferiority. These require distinct hypotheses, prespecified margins, and confidence intervals that satisfy the corresponding decision criteria. Effect estimates and confidence intervals are therefore more informative than a binary classification of results as significant or non-significant [43]. Likewise, a significant change within one group and a non-significant change within another do not establish a difference between treatments; that conclusion requires a direct between-group comparison.

It would nevertheless be inaccurate to describe all available studies as lacking a sample-size justification. The calculations in Pinckaers et al. [7] and van der Heijden et al. [20] addressed the detection of differences in MPS between protein conditions, whereas Lim et al. [19] based the calculation on the increase from postabsorptive to postprandial MPS. These designs answer different questions from whether two protein sources produce effects within a clinically acceptable margin. Small samples, attrition, and short observation periods add uncertainty, but the absence of an appropriate comparative hypothesis remains a separate limitation.

Differences between amino acid responses and MPS findings are not necessarily contradictory. Circulating amino acid concentrations, anabolic signaling, and tracer-derived synthesis rates measure different physiological processes over different sampling intervals [2,7,20,22,23,24,25,26]. An early leucine peak cannot substitute for a direct MPS measurement, and a signaling measurement at a selected time point cannot establish the magnitude of the integrated synthetic response. Meal composition, protein processing, amino acid composition, and the timing of sampling may help explain variation across studies [4,5,25,26], although the available comparisons do not isolate their respective contributions. The human–in vitro approach used by Salles et al. [26] provides mechanistic information about the activity of postprandial plasma, but it does not measure protein synthesis in human skeletal muscle.

Acute MPS responses establish a physiological effect under the conditions tested; they do not directly predict cumulative hypertrophy or strength gains. Integrated measurements over several days extend the observation period but still differ from long-term training outcomes [12,18]. The apparent discrepancy between acute amino acid differences and limited separation of training outcomes therefore remains unresolved. Possible explanations include dietary context, the size of the true comparative effect, and insufficient precision or duration. The current evidence cannot determine which explanation predominates.

Dietary context is particularly relevant when comparing supplement doses. The background diets and total protein intakes in the training and short-term MPS studies differed substantially [9,12,17,18]. A supplement added to a relatively low-protein diet does not test the same nutritional question as one added to an already higher-protein intake. Dose comparisons should therefore use actual protein content, accompanied where available by EAA and leucine provision, rather than powder mass alone. General protein recommendations provide context for interpreting these interventions [44,45], but the studies reviewed do not establish a universal pea-protein dose or leucine threshold. The possibility that differences between sources become more consequential at lower total protein intakes remains a hypothesis requiring direct testing.

Formulation studies must also be interpreted according to the contrast they actually test. Blends evaluate combined protein sources, while comparisons involving fortification or probiotics assess the contribution of those modifications [16,17,19,20,21]. A pea-supplemented dietary pattern involves additional differences in food and protein-source composition [18]. These studies can inform the use of the tested interventions without isolating the effect of pea protein itself. The linked Korzepa publications should also be treated as reports from the same trial rather than independent confirmation of a dietary effect [18,23].

**Figure 2:**
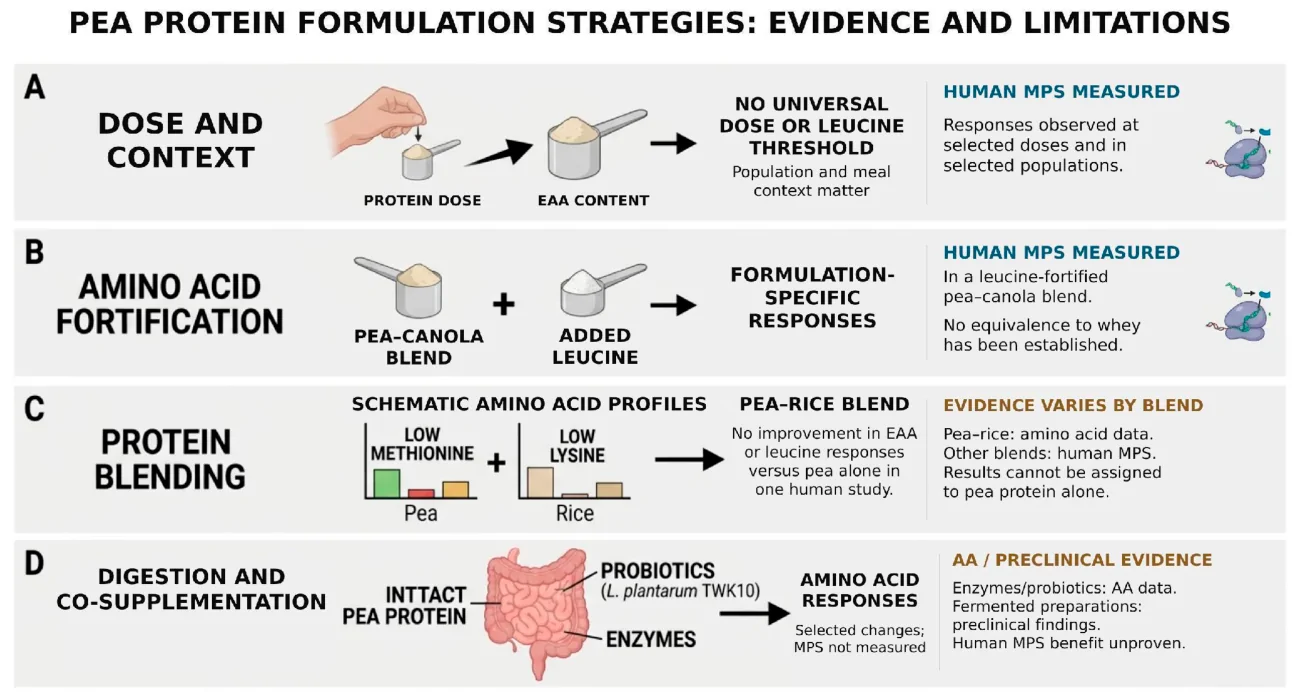
Pea protein formulation strategies and the evidence supporting their interpretation. (**A**) Dose and context: studies evaluating different pea protein doses indicate that responses depend on total protein and EAA provision, population characteristics, and meal context; no universal dose or leucine threshold has been established. (**B**) Amino acid fortification: leucine-fortified pea-based formulations have shown formulation-specific responses, but available data do not establish equivalence to whey protein. (**C**) Protein blending: blending pea protein with other plant proteins may modify amino acid profiles, although evidence varies across formulations and cannot be attributed to pea protein alone. (**D**) Digestion and co-supplementation: enzymes, probiotics, fermentation, and related strategies may influence amino acid responses, but evidence is limited and human MPS benefits remain unproven. The four panels retain the approaches considered in the review while distinguishing human MPS measurements, circulating amino acid responses, and preclinical findings. Dose should be interpreted with total dietary protein, EAA provision, population, and meal context; no universal dose or leucine threshold is established. Findings from blends and co-supplements apply to the formulations tested and do not establish equivalence to whey. AA, amino acids; EAA, essential amino acids; MPS, muscle protein synthesis.

There is no consistent basis for assuming that blending, fortification, or digestive adjuncts invariably improve pea-protein responses. The pea–rice comparison did not show significantly greater amino acid responses than pea alone [24], while matching selected aspects of amino acid composition did not eliminate differences in peripheral amino acid appearance in other formulations [25]. Human MPS measurements support the interpretation of particular fortified or blended interventions [19,20,21]; evidence for digestive adjuncts relies predominantly on circulating amino acid outcomes [27,28]. Their potential utility therefore depends on the formulation and endpoint assessed. Figure 2 distinguishes these approaches according to whether the supporting evidence concerns human MPS, amino acid responses, or preclinical mechanisms.

Exercise-recovery findings require a similarly outcome-specific interpretation. Changes in creatine kinase, myoglobin, or lactate dehydrogenase do not necessarily indicate faster restoration of strength or performance. The reviewed studies differ in exercise model, participant characteristics, supplementation schedule, and comparator, and biochemical and functional outcomes do not consistently agree [29,30,32,33]. A favorable biomarker comparison should therefore remain a conclusion about that marker and protocol. The available evidence does not support a general claim that pea protein accelerates functional recovery or that recovery responses are interchangeable with those following whey supplementation.

Preclinical evidence helps identify mechanisms and experimental conditions worth investigating, but its relevance to human efficacy is limited by species, aging models, dietary composition, and intervention design. Animal comparisons using intact pea protein address a different question from interventions involving inulin, mixed proteins, whole-food matrices, peptides, or leucine fortification [34,35,36,37,38,39,40]. These experiments should not be treated as interchangeable support for isolated pea protein. Their main contribution is to identify how formulation and physiological context may modify muscle-related responses and to guide subsequent human studies.

Peptide research introduces a further distinction between the activity of a preparation and that of an individual sequence. Findings obtained with peptide mixtures cannot be attributed to a specific peptide unless that contribution is tested directly [38,42]. Cell experiments with defined sequences provide more specific mechanistic evidence, but MTT-based readouts and changes in atrophy-related gene expression do not by themselves demonstrate muscle growth or reduced protein degradation [38,41]. Processing and hydrolysis may generate peptides with biological activity [6,46,47], and improved in vitro digestibility or transport may support further investigation [48]. Translation to an orally consumed intervention nevertheless requires evidence that the relevant sequences survive digestion, are absorbed, and reach their target tissues at biologically active concentrations [49,50].

In practice, pea protein can be considered a contributor to dietary protein intake when its composition and the overall diet meet the individual’s nutritional needs [1,4,51]. Product selection should account for actual protein and EAA provision, dietary preferences, and the intended use, with recommendations adapted to the individual rather than derived from a single comparative trial [52]. Evidence from older adults involves different dietary contexts and protein comparators [12,15,18], while much of the direct training evidence concerns relatively short interventions [8,9,10]. Applying these findings to highly trained athletes, women, and long-term use in older populations therefore requires caution.

This review has limitations that also constrain its interpretation. Previous systematic and pea-focused reviews provide complementary perspectives [13,14], whereas the present narrative synthesis was intended to distinguish intervention attribution and physiological endpoints. It does not provide a pooled estimate of comparative effects or a formal certainty rating. Screening was not conducted independently in duplicate, and standardized risk-of-bias tools were not applied. The grouping of studies by directness helps organize the synthesis [53], but it does not replace an assessment of methodological quality. Exclusion of grey literature may have contributed to publication bias. These limitations preclude treating the number of included reports as a measure of evidential strength. Additionally, standardized quantitative risk-of-bias scoring tools were not applied across the diverse study designs, which precludes a formal quantitative grading of overall evidence certainty and represents a methodological limitation of this narrative synthesis.

Future trials should first specify whether their purpose is to test an additional supplementation benefit, superiority, non-inferiority, or equivalence. An appropriate control, clinically justified margins where relevant, and sample-size calculations matched to the primary hypothesis are then needed. Longer interventions should assess muscle mass, strength, and performance, alongside clearly reported dietary protein and EAA intake, supplement composition, adherence, and attrition. Broader recruitment of women, older adults, and highly trained participants would improve applicability. Until such evidence is available, physiological responses to pea protein and improvements observed during training should remain distinct from claims about its independent benefit or comparative efficacy.

## 5. Conclusions

This review suggests that single-source pea protein isolate can support postprandial muscle protein synthesis and may accompany resistance-training adaptations under the conditions investigated in the available studies. However, the training trials do not consistently establish an additional benefit attributable to pea protein supplementation (Table 4). The evidence remains insufficient to establish superiority, equivalence, or non-inferiority relative to dairy proteins. The available studies also do not establish a universal effective dose or a consistent benefit for functional recovery.

Findings from pea-containing blends, fortified formulations, and digestive adjuncts remain specific to the interventions and outcomes assessed, while preclinical findings cannot establish efficacy in humans. Overall, pea protein isolate can contribute to dietary protein intake when its composition and the overall diet meet individual nutritional needs. Future long-term, adequately powered trials should distinguish additional supplementation benefits from differences between protein sources, using appropriate controls and prespecified hypotheses. Equivalence and non-inferiority trials require clinically justified margins, and broader recruitment of women, older adults, and trained athletes would improve applicability.

## Figures and Tables

**Table 1:** Longitudinal human studies evaluating pea-derived protein supplementation during structured exercise training.

Study	Country	Study Design	Registration	Population	Sample, *n*; Sex	Intervention	Comparator	Protocol	Outcomes Assessed
Babault et al., 2015 [8]	France	RCT, parallel-group	NCT02128516	Untrained men	137 analyzed (161 rand.); 100% M	Pea protein isolate (50 g/d: 2 × 25 g)	Whey protein concentrate (50 g/d); placebo	12-wk resistance training	Biceps thickness; 1-RM strength
Banaszek et al., 2019 [9]	USA	Randomized pilot trial	NR	HIFT-trained adults	15 (7 F, 8 M)	Pea protein (~49 g/d: 2 × 24.5 g)	Whey protein (~49 g/d)	8-wk HIFT	1-RM squat/deadlift; muscle thickness; IMTP peak force; body comp.
Singh et al., 2024 [10]	Canada	Triple-blind RCT, parallel	NCT04814225	Sedentary adults (30–59 y)	35 analyzed (50 rand.); 26 F, 24 M	Pea protein (20.0–22.5 g/d)	Whey protein isolate (20.0–21.1 g/d)	84-d resistance training	Composite muscle strength; endurance; body comp.
Santini et al., 2025 [17]	Brazil	Randomized clinical trial	NCT05710614	Healthy active adults (omnivorous)	44 analyzed (52 rand.); 100% M	Soy–pea blend (45 g/d: 3 × 15 g)	Whey protein (45 g/d)	12-wk lower-limb RT (3×/wk)	Lean mass (whole/appendicular/leg); mCSA; leg-press strength
Lee et al., 2024 [16]	Taiwan	Double-blind RCT, parallel	NR	Healthy adults	32 (16 F, 16 M)	Pea protein (20 g/d) + *L. plantarum* TWK10	Pea protein (20 g/d) alone	28-d resistance training (3×/wk)	Muscle thickness; 1-RM bench press; power; AA response

Abbreviations: 1-RM, one-repetition maximum; CFU, colony-forming units; HIFT, high-intensity functional training; IMTP, isometric mid-thigh pull; mCSA, muscle cross-sectional area; NR, not reported; RFD, rate of force development; RT, resistance training; WOD, workout of the day.

**Table 2:** Acute and short-term human studies evaluating pea-derived protein in relation to muscle protein synthesis, postprandial amino acid availability, anabolic signaling, and exercise recovery.

Study	Country	Study Design	Registration	Population	Sample, *n*; Sex	Intervention	Comparator	Protocol	Outcomes Assessed
Muscle protein synthesis (acute and integrated human evidence)									
Korzepa et al., 2025 (MPS) [18]	UK	Single-blind RCT, parallel	NCT05574205	Middle-to-older adults (50–70 y)	27	Plant-focused diet + pea protein (~1.0 g/kg/d total protein)	Animal-focused diet + whey protein (~1.0 g/kg/d total protein)	10-d diet + unilateral RT every 2nd d	Integrated myofibrillar MPS (trained/untrained); strength
Lim et al., 2024 [19]	Canada	Randomized crossover trial	NR	Healthy young adults	8 (4 F, 4 M)	Pea–canola blend (20 g protein, 88:12) ± leucine	Whey protein isolate (20 g protein)	Acute resting feeding study	Mixed-muscle FSR; postprandial aminoacidemia
McKendry et al., 2024 [12]	Canada	RCT, sequential dietary phases	NR	Healthy older men	31 (100% M)	Pea protein (50 g/d: 2 × 25 g)	Whey protein (50 g/d); collagen (50 g/d)	7-d baseline diet (0.8 g/kg/d) + 7-d supplement phase (1.3 g/kg/d)	Integrated myofibrillar MPS (FSR); anabolic signaling
Pinckaers et al., 2022 [21]	Netherlands	Double-blind RCT, parallel	NR	Active young men	24 (100% M)	Plant blend (30 g protein: wheat/corn/pea 50:25:25)	Milk protein (30 g protein)	Acute tracer feeding study	Postprandial myofibrillar MPS; plasma AAs; insulin
Pinckaers et al., 2024 [7]	Netherlands	Double-blind RCT, parallel	NTR6548	Active young men (18–35 y)	24 (100% M)	Pea protein concentrate (30 g protein)	Milk protein concentrate (30 g protein)	Acute 5-h tracer study (fasted, resting)	Postprandial myofibrillar MPS; plasma AA availability
van der Heijden et al., 2024 [20]	UK	Double-blind randomized crossover	NCT06129513	Resistance-trained young adults	10 (2 F, 8 M)	Plant blend (32 g protein: pea/rice/canola 40:40:20)	Whey protein (32 g protein)	Acute post-exercise feeding (4-h sampling)	Post-exercise myofibrillar MPS; plasma AAs
West et al., 2023 [11]	UK	Double-blind RCT, parallel	NCT04894747	Resistance-trained young adults	33 (9 F, 24 M)	Pea protein (25 g); pea–mycoprotein blend (25 g, 61:39)	Mycoprotein (25 g)	Acute post-exercise feeding (4-h sampling)	Post-exercise myofibrillar MPS; plasma AAs
Postprandial amino-acid availability and anabolic signaling									
de Marco Castro et al., 2022 [25]	Ireland	Single-blind crossover trial	NR	Older adults (≥65 y)	9	Pea-containing plant–fiber products (2.8 g leucine)	Whey protein + fiber	Acute 3-h postprandial sampling (4 test meals)	Peripheral AA appearance; leucine/BCAA iAUC; insulin
Korzepa et al., 2025 [18] (aminoacidemia)	UK	Single-blind RCT, parallel	NCT05574205	Active adults (50–70 y)	22 analyzed (27 recruited)	Low-protein breakfast + pea protein isolate (~0.13 g/kg)	Low-protein breakfast + whey protein concentrate (~0.13 g/kg)	Acute 180-min postprandial blood sampling	Plasma TAA, EAA, leucine iAUC; appetite
Lanng et al., 2023 [22]	Denmark	Single-blind RCT, parallel	NCT04633694	Healthy young men	50 (100% M)	Pea protein (0.25 g/kg FFM)	Whey protein (0.25 g/kg FFM); cricket protein (0.25 g/kg FFM)	Acute post-exercise feeding; biopsies at 0 & 3 h	Plasma EAA/BCAA/leucine kinetics; mTORC1 phosphorylation
Paulussen et al., 2024 [27]	USA	Double-blind crossover RCT	NR	Healthy adults	24 (12 F, 12 M)	Pea protein isolate (20 g protein) + microbial proteases	Pea protein isolate (20 g protein) + placebo	Acute 5-h postprandial blood sampling	Plasma TAA, EAA, BCAA, leucine AUC; glucose/insulin
Rathi et al., 2024 [28]	India	Double-blind crossover RCT	NR	Healthy young adults (18–35 y)	15 (100% M)	Pea protein (30 g/d) + enzyme–probiotic blend	Pea protein (30 g/d) + placebo	Two 15-d supplementation periods (30-d washout)	Plasma AA AUC/Cmax; gut microbiome; urinary/fecal N
Rogers et al., 2024 [24]	UK	Randomized crossover trial	NR	Healthy young adults	10 (6 F, 4 M)	Pea isolate (0.3 g/kg); pea–rice blend (0.3 g/kg, 1:1)	Whey protein concentrate (0.3 g/kg)	Acute 4-h postprandial blood sampling	Plasma EAA, leucine iAUC & Cmax; appetite
Salles et al., 2024 [26]	France	Randomized crossover trial	NCT06381869	Young (20–28 y) and older men (65–81 y)	30 (15 young, 15 older)	Pea protein (0.41 g/kg) ± standardized meal	Whey protein (0.41 g/kg) ± standardized meal	4 acute conditions; ex vivo plasma C2C12 myotube incubation	Postprandial aminoacidemia; C2C12 myotube protein synthesis
Exercise-recovery outcomes									
Coutinho et al., 2014 [29]	Brazil	Controlled parallel trial	NR	Military personnel (soldiers)	23 analyzed	Carbohydrate (0.8 g/kg/h) + pea protein (0.2 g/kg/h)	Carbohydrate alone (0.8 or 1.0 g/kg/h)	Strenuous exercise; dosing at 0, 60, 120 min post-exercise	CK, LDH; maximal isometric strength; recovery
Hosseinzade et al., 2022 [31]	Iran	Controlled comparative trial	NR	Healthy young men	30 (100% M)	Pea protein isolate (0.3 g/kg pre- & post-exercise + bedtime)	Whey protein isolate (matched dose); placebo	Single 60-min HIFT session; recovery monitoring	DOMS; CK, LDH; physical fitness performance
Loureiro et al., 2023 [33]	Brazil	Double-blind crossover RCT	NR	Male U-20 soccer players	12 (100% M)	Pea protein concentrate (0.5 g/kg/d)	Whey protein (0.5 g/kg/d)	10-d supplementation surrounding soccer match (72-h tracking)	CK, lactate; countermovement jump; DOMS; metabolomics
Nieman et al., 2020 [30]	USA	Double-blind RCT, parallel	NR	Non-athletic men (18–55 y)	92 (100% M)	Pea protein isolate (0.9 g/kg/d: 3 × 0.3 g/kg)	Whey protein isolate (0.9 g/kg/d); water control	90-min eccentric exercise + 5-d recovery period	CK, myoglobin; DOMS; performance recovery
Spoelder et al., 2023 [32]	Netherlands	Double-blind RCT, parallel	NL9499	Active older adults (≥60 y)	45 analyzed (9 F, 36 M)	Pea protein (25 g/d)	Whey protein (25 g/d); placebo	13-d supplementation surrounding 20–30 km walk	CK, LDH; muscle soreness; handgrip/leg strength

The two Korzepa reports share trial registration NCT05574205; they are separate publications with potentially overlapping participants. Abbreviations: AA, amino acid; AUC, area under the curve; BCAA, branched-chain amino acid; BW, body weight; Cmax, maximum concentration; CRP, C-reactive protein; DOMS, delayed-onset muscle soreness; EAA, essential amino acid; FSR, fractional synthetic rate; IAA, indispensable amino acid; iAUC, incremental area under the curve; MPS, muscle protein synthesis; mTORC1, mechanistic target of rapamycin complex 1; NR, not reported; TAA, total amino acids; Tmax, time to maximum concentration.

**Table 3:** Characteristics of preclinical and in vitro studies investigating pea-derived proteins in relation to skeletal muscle outcomes.

Study	Country	Experimental Model/Sample	Intervention/Formulation	Comparator	Duration/Protocol	Outcomes Assessed
Berrazaga et al., 2020 [37]	France	Old Wistar rats (22 mo; *n* = 43)	Split-pea pasta (79% pea, 21% wheat)	Casein; soluble milk proteins; faba/lentil pasta	6-wk dietary intervention	Whole-body N retention; skeletal-muscle MPS
Dijk et al., 2025 [40]	Netherlands	Aged C57BL/6J mice (25 mo; *n* = 48)	Pea–soy blend (51:49) ± leucine (12% free leu)	Whey protein; fasted control	Acute oral gavage (~70 mg protein)	Skeletal-muscle MPS; plasma AA kinetics
Feng et al., 2021 [42]	China	Kunming mice (*n* = 150)	Pea peptides (<2 kDa: 100, 200, 400 mg/kg/d)	Isolated pea protein (200 mg/kg/d); water control	30-d daily gavage + swim test	Swimming endurance; BUN, BLA, glycogen; LDH
Jia et al., 2022 [38]	China	Sprague–Dawley rats (8 wk; *n* = 30)	Pea peptides (0.4 g/kg/d) + resistance exercise	Exercise alone; non-exercise control	8-wk resistance training (3×/wk)	Muscle mass/strength; mTOR/Akt signaling
Kim et al., 2025 [41]	Korea	C2C12 myotubes (dexamethasone)	Fermented pea peptides (IQRPVKEL, IENPVKEL)	Dexamethasone control; vehicle control	In vitro exposure	Muscle atrophy markers (MuRF-1, FoxO3); Sirt1/AMPK
Lépine et al., 2023 [36]	France	Rats on high-fat, high-sucrose diet	Wheat–pea protein blend	Milk protein	Dietary intervention	Tissue protein anabolism; metabolic health
Özdemir et al., 2023 [39]	Turkey	Exercised rats (*n* = 24)	Pea–rice blend (1:1, 0.465 g/kg) ± amylopectin/Cr	Exercise-only control	Acute post-exercise gavage (1-h sampling)	Myofibrillar MPS; mTOR, S6K1, 4E-BP1 phosphorylation
Salles et al., 2021 [34]	France	Old Wistar rats (20 mo; *n* = 30)	Diet with 14% pea protein	Diet with 14% casein; diet with 14% whey	16-wk dietary intervention	Plantaris MPS; p70S6K phosphorylation; body comp.
Salles et al., 2023 [35]	France	Old Wistar rats (20 mo; *n* = 26)	Diet with 14% pea protein + 7.5% inulin	Diet with 14% pea protein alone	16-wk dietary intervention	Fast-twitch muscle mass; mitochondrial activity

Abbreviations: 4E-BP1, eukaryotic translation initiation factor 4E-binding protein 1; BLA, blood lactic acid; BUN, blood urea nitrogen; BW, body weight; EFPP, enzyme-modified and fermented pea protein; FSR, fractional synthetic rate; GSH-Px, glutathione peroxidase; IL-6, interleukin-6; LDH, lactate dehydrogenase; MDA, malondialdehyde; MPS, muscle protein synthesis; mTOR, mechanistic target of rapamycin; S6K1/p70S6K, ribosomal protein S6 kinase beta-1; sIgA, secretory immunoglobulin A; SOD, superoxide dismutase; TNF-α, tumor necrosis factor-alpha.

**Table 4:** Key take-home messages summarizing the evidence on pea-derived protein supplementation across physiological domains, clinical implications, and research gaps.

Domain	Key Finding	Practical Implication
Acute MPS	Pea protein stimulated MPS in selected acute human studies.	Supports acute MPS under studied conditions; equivalence to dairy is unproven.
Amino acid kinetics	Whey often produced higher early EAA and leucine responses; pea–rice did not improve responses over pea alone in one study.	Amino acid responses do not establish MPS or a general dose threshold.
Training adaptations	Improvements occurred during training, but pea’s added effect and equivalence to whey remain unestablished.	Pea protein may accompany training adaptations under studied conditions.
Formulations	Blends and co-supplements changed some amino acid responses but do not isolate pea’s effect.	Interpret findings for the tested formulation.
Recovery	Biomarker results varied; functional benefits were not consistent.	Biomarker changes alone do not show functional recovery.
Evidence gaps	Trials vary and were not designed to test equivalence or non-inferiority.	Larger, longer comparative trials are needed.

Abbreviations: EAA, essential amino acids; MPS, muscle protein synthesis.

## Data Availability

No new data were created or analyzed in this study. Data sharing is not applicable to this article.

## Supplementary Materials

The following supporting information can be downloaded at: https://www.mdpi.com/article/10.3390/nu18213612/s1. Figure S1: Flow diagram of the study selection process. Flowchart detailing the literature search, screening, and study selection phases.

## Abbreviations

The following abbreviations are used in this manuscript:

1-RM	one-repetition maximum
4E-BP1	eukaryotic translation initiation factor 4E-binding protein 1
AA	amino acid
AUC	area under the curve
BCAA	branched-chain amino acid
BLA	blood lactic acid
BUN	blood urea nitrogen
BW	body weight
CFU	colony-forming units
Cmax	maximum concentration
CRP	C-reactive protein
DOMS	delayed-onset muscle soreness
EAA	essential amino acid
EFPP	enzyme-modified and fermented pea protein
FSR	fractional synthetic rate
GSH-Px	glutathione peroxidase
HIFT	high-intensity functional training
IAA	indispensable amino acid
iAUC	incremental area under the curve
IL-6	interleukin-6
IMTP	isometric mid-thigh pull
LDH	lactate dehydrogenase
mCSA	muscle cross-sectional area
MDA	malondialdehyde
MPS	muscle protein synthesis
mTOR	mechanistic target of rapamycin
mTORC1	mechanistic target of rapamycin complex 1
NR	not reported
p70S6K/S6K1	ribosomal protein S6 kinase beta-1
RFD	rate of force development
RT	resistance training
sIgA	secretory immunoglobulin A
SOD	superoxide dismutase
TAA	total amino acids
Tmax	time to maximum concentration
TNF-α	tumor necrosis factor-alpha
WOD	workout of the day
