# Supplementary material for: Pea-Derived Protein Supplementation in Sports and Exercise Nutrition: A Narrative Review of Muscle Protein Synthesis and Training Adaptations
Source: Nutrients. 2026 Oct 30;18(21):3612. doi: 10.3390/nu18213612 (PMC13765204; doi:10.3390/nu18213612)
Supplement: Supplementary file 1 [file nutrients-18-03612-s001.zip › nutrients-4664645-supplementary.pdf]

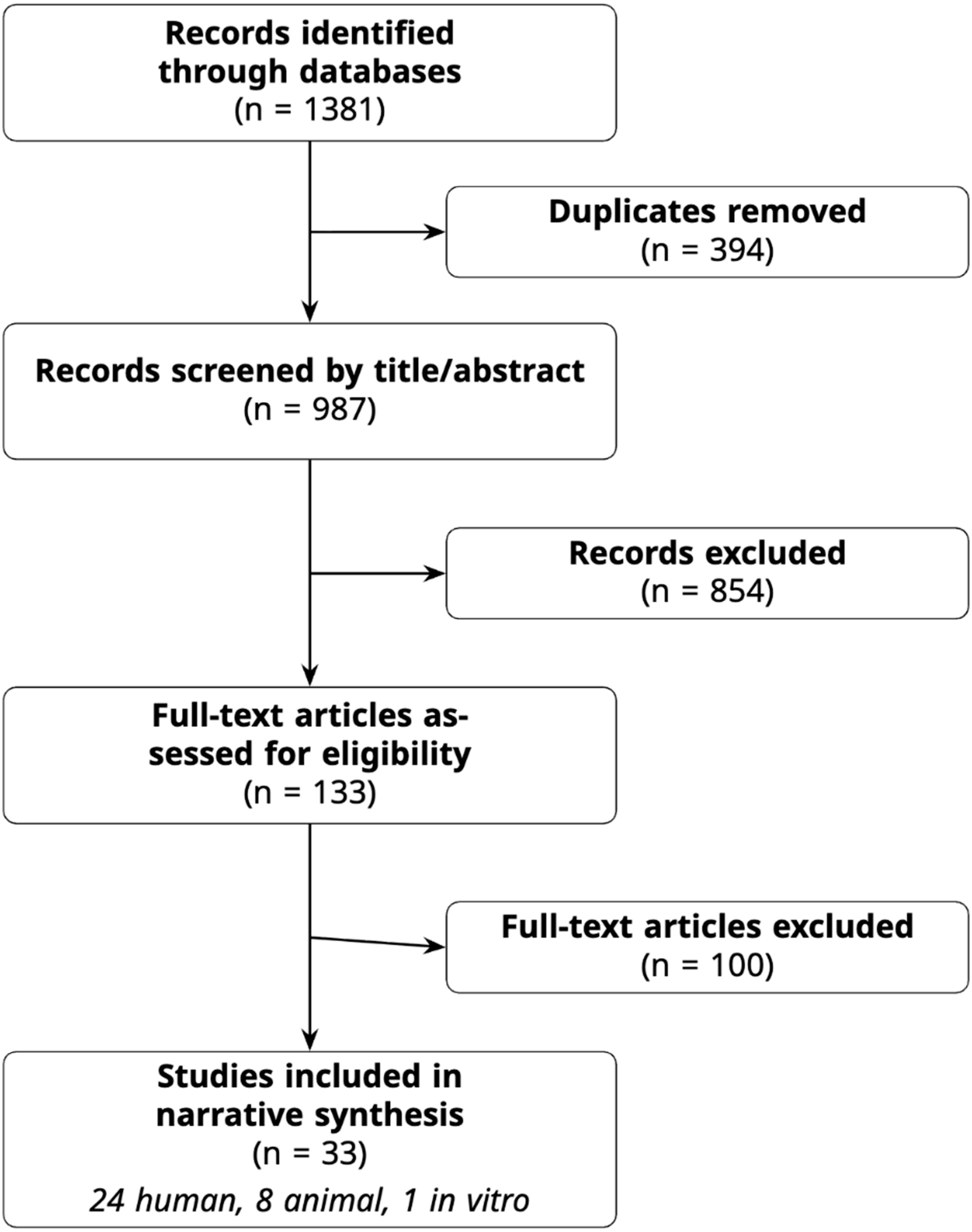
Records identified through databases
(n = 1381)
Duplicates removed
(n = 394)
Records screened by title/abstract
(n = 987)
Records excluded
(n = 854)
Full-text articles assessed for eligibility
(n = 133)
Full-text articles excluded
(n = 100)
Studies included in narrative synthesis
(n = 33)
24 human, 8 animal, 1 in vitro

Supplementary Figure S1. Flow diagram of the study selection process. Flowchart detailing the literature search, screening, and study selection phases.
